# A New Anesthetic, Remimazolam, Is Useful in the Management of Anesthesia in Patients with Liver Cirrhosis

**DOI:** 10.1155/2022/9268454

**Published:** 2022-05-07

**Authors:** Anna Onoda, Yasuyuki Suzuki

**Affiliations:** ^1^Center for Medical Education and Training, Saiseikai Matsuyama Hospital, Matsuyama, Japan; ^2^Department of Anaesthesiology, Saiseikai Matsuyama Hospital, Matsuyama, Japan; ^3^Department of Pharmacology, Ehime University Graduate School of Medicine, Matsuyama, Japan; ^4^Research Division, Saiseikai Research Institute of Health Care and Welfare, Tokyo, Japan

## Abstract

**Background:**

Management of general anesthesia in patients with liver cirrhosis is challenging because it is difficult to maintain the circulation and concentration of anesthetics within a safe range. Unlike many other anesthetics, which are metabolized by cytochrome P450 enzymes, remimazolam is metabolized by carboxylesterase. In a liver cirrhosis model, cytochrome P450 activity is suppressed by approximately 30%; however, carboxylesterase activity is maintained at approximately 60%. Also, remimazolam is less likely to inhibit circulation. A 77-year-old woman was scheduled to undergo laparoscopic cholecystectomy. The patient was diagnosed with Child-Pugh B liver cirrhosis due to type C viral hepatitis. General anesthesia with remimazolam stabilized the intraoperative circulation and resulted in rapid postoperative awakening.

**Conclusion:**

We report a case in which a patient with Child-Pugh B cirrhosis was safely managed under general anesthesia using remimazolam during laparoscopic cholecystectomy.

## 1. Introduction

In patients with liver cirrhosis, anesthesia for laparoscopic cholecystectomy is difficult [1–3]. Patients with cirrhosis have difficulty maintaining circulatory dynamics due to instability in circulating plasma volume caused by decreased albumin production in the liver and changes in the status of the circulation, such as congestion due to fluid overload or low cardiac output due to low fluid [4]. Some reports highlighted increasing morbidity and mortality in patients with liver cirrhosis after laparoscopic cholecystectomy [5]. To maintain stable circulatory dynamics, anesthetics with minimal circulatory depressant effects should be selected [3].

Moreover, the damaged liver inhibits drug metabolism, which causes delayed emergence from anesthesia. Most drugs are metabolized by cytochrome P450 enzymes (CYP). Another metabolic enzyme of interest is human carboxylesterase (HCE), which plays two roles in terms of drug metabolism [6]. It activates prodrugs, or it inactivates drugs by hydrolyzing ester bonds [6]. HCE1 metabolizes remimazolam, which is different from many other sedative drugs, including propofol and midazolam [7].

Herein, we report a case of safe management of general anesthesia during laparoscopic cholecystectomy in a patient with liver cirrhosis using remimazolam.

## 2. Case Presentation

A 77-year-old woman (height: 144 cm; weight: 49.6 kg) was scheduled to undergo laparoscopic cholecystectomy. The patient was diagnosed with liver cirrhosis at 72 years due to chronic type C viral hepatitis. On physical examination, the abdomen was flat and soft. Swelling of her legs and hands was noted, and jaundice of the skin was mild. A neurological examination was completely unremarkable, and there was no evidence of encephalopathy. Hematological tests showed an increase in alkaline phosphatase and bilirubin (alkaline phosphatase: 428 IU·mL^−1^ and total bilirubin: 2.19 mg·dL^−1^), a low albumin concentration (3.5 g·dL^−1^), and a low platelet count (3.0 × 10^4^ *μ*L^−1^). Aspartate transaminase and alanine aminotransferase were within normal ranges. Abdominal computed tomography showed slight ascites in the abdomen, which seemed to be a physiological phenomenon. Based on these observations, the patient's Child-Pugh score was 7 points (grade B). There was no evidence of cardiac hypofunction with echocardiography or electrocardiography. The chest X-ray did not reveal cardiac expansion. We planned to use remimazolam in the hope of rapid metabolism and stabilization of circulatory dynamics. Upon entering the operating room, the patient's blood pressure, pulse, and oxygen saturation (in room air) were 184/91 mmHg, 89 min^−1^, and 100%, respectively.

Anesthesia was induced with remimazolam (3 *μ*g·kg^−1^·h^−1^), remifentanil (0.1 *μ*g·kg^−1^·h^−1^), 250 *μ*g of fentanyl, and 30 mg of rocuronium. On falling asleep (guided by the bispectral index (BIS) value), the remimazolam administration rate was reduced to 0.8 mg·kg^−1^·h^−1^ and was maintained at around 0.5 mg·kg^−1^·h^−1^ during the operation. For postoperative analgesia, we performed an echocardiography-guided transabdominal plane block and administered a 60-mg dose of ropivacaine.

**Figure 1:**
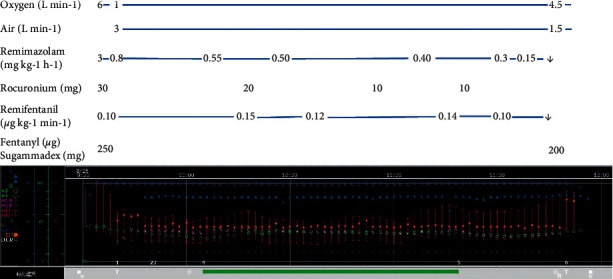
Anesthetic chart of this case. The anesthetic chart recorded by Prescient ORFujifilm Medical Corporation, Tokyo, Japan. T1: body temperature; HR: heart rate; PR: pulse rate; NIBP: noninvasive blood pressure; ART: arterial pressure (invasive); RR: respiratory ratio; SpO2: oxygen saturation; BIS: Bispectral Index; EtCO2: end-tidal carbon dioxide; ■: in operation room and out operation room; ×: start of anesthesia and end of anesthesia; T: intubation and extubation; ◎: start of operation and end of the operation; R: Sugammadex reverse rocuronium.

During the operation, the mean blood pressure was maintained at around 70 mmHg. Thus, we did not use vasopressor agents, such as ephedrine. The BIS value was approximately 45 during the operation. The surgery took 83 minutes, and the volume of blood loss was approximately 10 ml. We observed a smooth awakening 5 minutes after the operation and an excellent postoperative course (Figure 1). Immediately after the surgery, no antagonist was used because her vitals were stable, and she did not fall into a sedated state again. The patient passed without any postoperative complications, and we found no worsening of her liver function. She was discharged without any problems on postoperative day 5.

## 3. Discussion

We reported a patient with liver cirrhosis in whom remimazolam was used to achieve stable circulatory dynamics and rapid awakening after general anesthesia for laparoscopic cholecystectomy.

Like midazolam, remimazolam is a benzodiazepine anesthetic. Remimazolam has a similar structure to midazolam, but the former has an ester-linked side chain to the diazepine ring, making it an ultra-short-acting intravenous formulation rapidly metabolized by HCE1 in the liver. Another essential feature of remimazolam is that the hepatic drug-metabolizing enzyme, CYP, is not involved in its metabolism, while propofol (a common sedative drug for total intravenous anesthesia) and midazolam (a benzodiazepine sedative) are both metabolized by CYP. These enzymes are widely distributed throughout the body, particularly in the liver. Because CYP expression in the liver is suppressed in patients with liver cirrhosis, propofol and midazolam metabolism decrease when administered to patients with liver damage [8]. Drug elimination with significant hepatic metabolism worsens as liver cirrhosis progresses. Albarmawi et al. reported that, in patients classified as Child-Pugh B and C, unbound midazolam clearance was reduced to 23% and 14%, respectively. Prolonged sedation was observed in patients who received high doses of midazolam because the half-life of midazolam is prolonged in patients with higher Child-Pugh scores [9].

In mammals, carboxylesterase enzymes are classified into five families, with HCE1 and HCE2 playing a key role in drug metabolism [10]. There are no reports on changes in carboxylesterase enzymes in patients with cirrhosis, but there is one report on ex vivo experiments using cirrhotic model cells. Plated human hepatocytes were treated with 50 ng/mL interleukin-6 (a cytokine that triggers hepatocyte inflammation in cirrhosis) for 24 hours. They studied changes in HCE1, human carboxylesterase 2, and CYP 3A4 mRNA. With interleukin-6 treatment, HCE1 mRNA decreased by 20% to 40% compared with the 64% to 99% decline in CYP3A4 mRNA [11]. We speculate that there may be sufficient carboxylesterase activity to metabolize clinical doses of remimazolam, even in patients with cirrhosis. Recently, Stöhr et al. reported that the maximum observed concentration was independent of hepatic function in a study of bolus doses of remimazolam in patients with abnormal liver function [12]. Also, it is vital that the metabolites have no pharmacological activity, which might not cause awakening delay. However, during general anesthesia, remimazolam is administered as a continuous intravenous infusion not a bolus dose, so it cannot be said that remimazolam is suitable for anesthesia management in patients with cirrhosis based on these study data alone. We believe that a clearer answer will be obtained when the study of continuous administration is conducted in the future.

Remimazolam is also excellent in the point of stabilizing circulatory dynamics during surgery. In liver cirrhosis, the liver cannot produce an adequate amount of albumin, which causes a decrease in the plasma colloid osmotic pressure. Thus, water cannot be retained in blood vessels, leading to ascites in the abdominal cavity. Moreover, increased portal pressure and nitric monoxide production cause dilation of peripheral arteries and decreased arterial blood volume. Thus, activation of the renin-angiotensin-aldosterone system, the sympathetic nervous system, and vasopressin cause salt and water retention, increasing circulating blood volume. This compensation depends on the degree of liver damage, so circulatory dynamics in patients with liver cirrhosis is extremely unstable [13].

Remimazolam does not have a robust circulatory depressant effect [14]. In a comparative study with propofol, intraoperative hypotension frequency was significantly lower when managed with remimazolam [15]. In studies comparing remimazolam with midazolam, the latter of which is proven to have a minimal circulatory depressant effect, the remimazolam's effect on circulatory dynamics was comparable to that of midazolam [16]. Thus, remimazolam may be useful in the anesthetic management of patients with liver cirrhosis who are prone to circulatory instability.

Although we experienced an interesting case, this study has several limitations. In this report, BIS values were reduced at a much lower dosage than that indicated in the package insert. Our results show that anesthesia maintenance tends to be achieved with relatively low doses of remimazolam, except in young patients. In such cases, anesthesia management is also possible with a small amount of remimazolam. While it is possible that the drug dosage of remimazolam in clinical trials was set too high, the possibility that the drug may affect drug metabolism when used in patients with cirrhosis cannot be dismissed. Indeed, the drug package insert cautions that drug metabolism is delayed in patients classified as Child-Pugh C. As the use of remimazolam in patients with cirrhosis increases in the future, we expect that the most appropriate dosage will become clear.

In terms of circulatory dynamics, although the patient was classified as Child-Pugh B, the circulatory system was stable before surgery. In addition, although laparoscopic cholecystectomy is concerned about the effect of insufflation on circulatory dynamics, the amount of blood loss is usually small, and the effect on circulatory dynamics is relatively low. Minimally invasive surgery may have been a factor in the successful management of this case. However, caution may be necessary for patients with more severe liver cirrhosis and preoperative circulatory instability.

## 4. Conclusions

Remimazolam is a good choice for anesthesia in patients with liver cirrhosis to stabilize circulation and achieve prompt awakening. It is hoped that the safety of remimazolam will be confirmed in the future as its use increases in patients with more severe cirrhosis and as drug concentrations in the blood are measured.

## Abbreviations

CYP, P450: Cytochrome P450
HCE: Human carboxylesterase
BIS: Bispectral Index.
